# Pulmonary Contusions in a Collegiate Football Player With Same-Game Return-to-Play

**DOI:** 10.7759/cureus.79752

**Published:** 2025-02-27

**Authors:** Robert Rollins, Joshua Altman, Kelsey Diemer, Andrew Smith, James R Clugston, Paul Silvestri, Tony Hill, Donavon White, Sarah Chrabaszcz

**Affiliations:** 1 Department of Emergency Medicine, University of Florida College of Medicine, Gainesville, USA; 2 Department of Community Health and Family Medicine, University of Florida College of Medicine, Gainesville, USA; 3 University Athletic Association, University of Florida, Gainesville, USA

**Keywords:** blunt thoracic trauma, prompt diagnosis, pulmonary contusions, return to sport, thoracic imaging

## Abstract

Pulmonary contusions are relatively common lung parenchymal injuries associated with high-energy thoracic trauma but have rarely been reported in sports participation. The most common symptoms include dyspnea and hemoptysis, but severe cases may develop hypoxemia and acute respiratory distress syndrome. Diagnosis is confirmed with thoracic imaging and treatment is supportive care, with most pulmonary contusions resolving within a week. Limited information exists regarding return-to-play guidelines in athletes with pulmonary contusions. We present a case of a collegiate football player who sustained right-sided chest wall trauma during competition and was diagnosed with small bilateral pulmonary contusions in a unique coup-contrecoup distribution, identified via chest computed tomography (CT). The athlete had rapid symptom resolution and was able to return to play during the same competition. No previous reports have described immediate diagnosis and return to play within the same game.

## Introduction

Pulmonary contusions are relatively common lung injuries associated with high-energy blunt thoracic trauma [1]. Clinical symptoms most commonly include cough, hemoptysis, and dyspnea which may be mild or asymptomatic in cases of small contusions, with larger, more severe contusions possibly leading to the development of hypoxemia, acute respiratory distress syndrome (ARDS) or pneumonia [2,3]. Diagnosis is confirmed using thoracic imaging, most commonly chest radiographs or computed tomography (CT) scans of the chest [4]. While these injuries are seen commonly in high-energy trauma, they have rarely been reported in sports participation, with limited case reports existing that describe diagnoses of pulmonary contusions during an athletic contest. We present a case of small bilateral pulmonary contusions diagnosed in a collegiate football player during competition with same-game return-to-play.

This article was previously presented as an abstract at the 2023 American Medical Society for Sports Medicine (AMSSM) Annual Meeting in April 2023.

## Case presentation

A 22-year-old male collegiate football player was struck by an opponent’s helmet on the right anterior chest wall and right upper quadrant (RUQ) of the abdomen early in the first half during a competition. He fell to the ground and was evaluated by athletic trainers at which time he complained of shortness of breath, right lower chest wall and RUQ pain. While walking off the field he had an episode of small volume hemoptysis. On the sideline his heart rate was 150 bpm, and oxygen saturation was 98-99% on room air. He was taken to the training room for further evaluation where he complained of right anterior chest and upper abdominal pain as well as postural dizziness. An extended focused assessment with sonography for trauma (eFAST) was negative for intraabdominal free fluid, pericardial effusion, or pneumothorax. Despite resting and resolution of hemoptysis, he continued to feel lightheaded with a heart rate of 130 bpm and was taken to the emergency department (ED) for further evaluation.

In the ED, initial vitals were blood pressure 157/89, pulse 97 beats/minute, respiratory rate 14 breaths/minute, temperature 36.4C, and oxygen saturation 99% on room air. On physical exam he was well-appearing and in no acute distress. Lungs were clear to auscultation bilaterally. There was no tachypnea, accessory muscle usage, respiratory distress, or retractions. The chest wall had no deformities, tenderness, or crepitus. No paradoxical rib motion was present. A cardiovascular exam revealed a regular rhythm and normal pulses. There was mild RUQ tenderness to palpation but no abdominal distention or guarding. The remainder of the exam was unremarkable.

**Figure 1:**
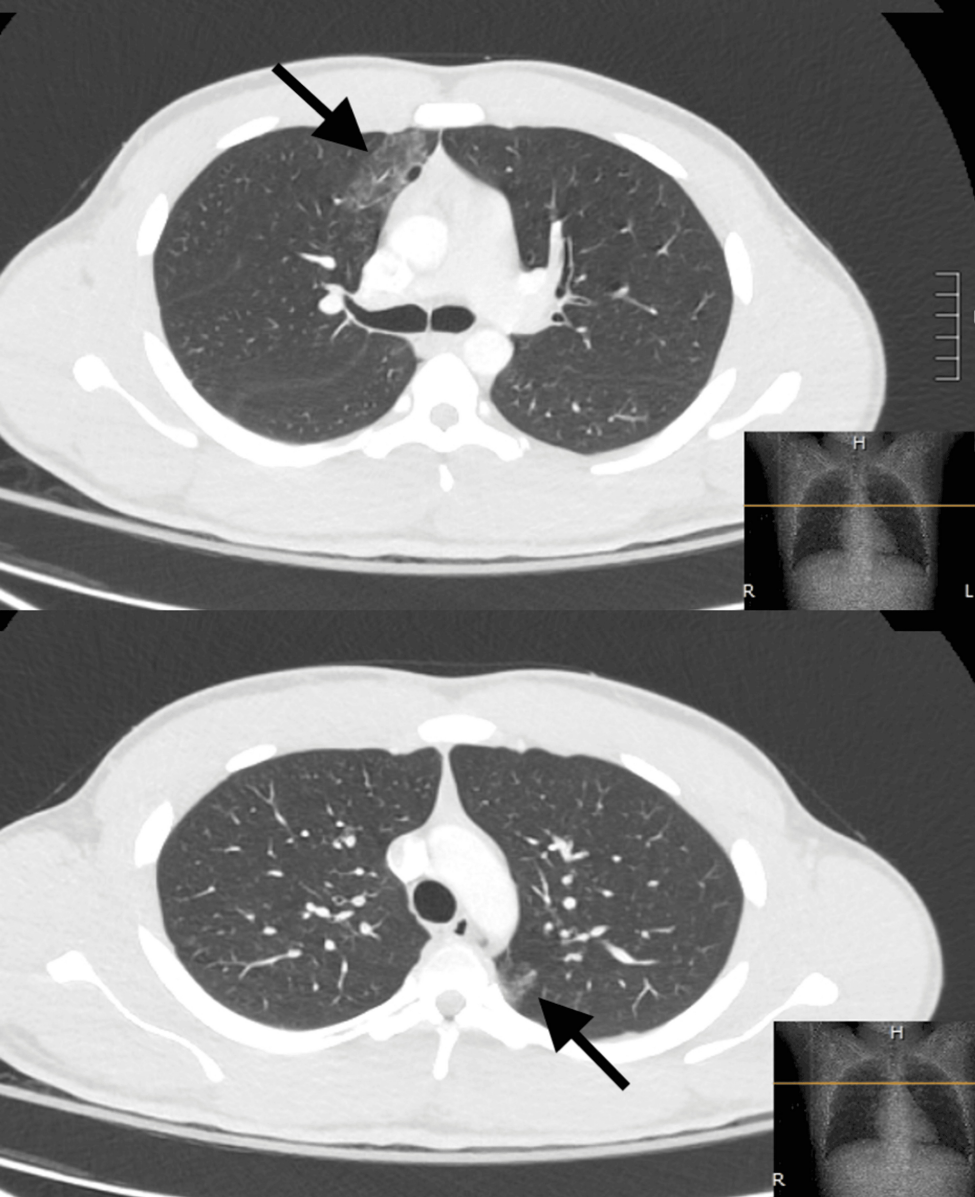
Computed tomography (CT) scan of the chest Axial images seen in lung windows from computed tomography (CT) scan of the chest with intravenous contrast enhancement revealing small ground glass opacities suggestive of pulmonary contusions (arrows).

Point-of-care labs were obtained as shown in Table 1. Chemistries and venous blood gas were unremarkable. A CT scan of the chest, abdomen, and pelvis was obtained (Figure 1) revealing a few small bilateral ground glass opacities suggestive of pulmonary contusions involving the right middle lobe, the superior segment of the right lower lobe, and the superior lingular segment of the left upper lobe suggesting a coup/contrecoup mechanism. There were no pneumothoraces, hemothoraces, rib fractures, or intraabdominal injuries seen.

**Table 1:** Point-of-care laboratory results PT: Prothrombin time; INR: international normalized ratio

Lab Test	Patient’s Results	Reference Range
Hemoglobin	17 g/dl	12-18 g/dl
Hematocrit	49% packed cell volume	39-49% packed cell volume
Lactate	2.88 mmol/L	0.3-1.5 mmol/L
PT	12.9 seconds	9.4-12.4 seconds
PT-INR	1.1	0.8-1.2

The patient remained hemodynamically stable in the ED with resolution of symptoms including pain and hemoptysis. He ambulated without desaturation and was subsequently discharged. With his rapid improvement in clinical symptoms, stable vital signs, resolution of hemoptysis, and pain he was allowed to return to the game. He successfully completed the game without any worsening symptoms. Following the game, he mentioned a heightened urge to cough with deep inspiration. The athlete rested for four days following the competition due to a planned platelet-rich plasma injection the next day for a previously diagnosed extremity injury. Four days after the game, the patient reported a complete resolution of cough. He participated in practice six days following his injury with no symptoms or complications.

## Discussion

Pulmonary contusions are bruises to the lung parenchyma most commonly due to blunt shearing forces resulting in edema and alveolar hemorrhage, which can result in impairment of physiologic function of the affected lung tissue [1]. The most common symptoms include dyspnea and hemoptysis [2]. Symptoms typically begin within hours of injury and peak around 72 hours, with severe cases potentially leading to hypoxemia, acute respiratory distress syndrome (ARDS), and pneumonia [3]. Most pulmonary contusions resolve within seven days from onset [3].

Chest radiographs are the primary screening examinations performed in thoracic trauma because they are relatively inexpensive, noninvasive, and can be performed rapidly at the bedside [4]. Characteristic radiograph features include focal patchy or diffuse nonsegmental hazy airspace opacities which may become consolidative when bleeding is extensive [4]. Small contusions may not be visible on standard radiographs in the first six hours [4]. Thoracic CT has a much higher sensitivity (with estimates approaching 100%) [3], aids in detection immediately after injury, is predictive of the need for mechanical ventilation [3], and evaluates for concomitant traumatic thoracic injuries. On CT imaging, lung contusions typically appear as regions of ground-glass opacities with subpleural sparing and do not follow lobar or segmental boundaries due to parenchymal insult occurring at the location of energy transfer rather than following an endobronchial route as seen in infectious processes [4].

Hypoxemia is a key predictor of outcome and is congruent with the degree of lung injury [3]. Larger contusions, often defined as greater than 20% of total lung volume, also have been shown to carry an increased risk of pulmonary complications [5]. Minor or occult contusions may be asymptomatic or with mild symptoms and typically require minimal supportive care [4]. Larger pulmonary contusions may result in impairment in gas exchange and lung compliance leading to hypoxemia, dyspnea, tachypnea, and tachycardia with more severe injuries potentially leading to pneumonia or ARDS. More severe contusions may require aggressive pain control, supplemental oxygen and respiratory support, fluid resuscitation, pulmonary hygiene, and hemodynamic monitoring, with increasing amounts of required support proportional to the degree and severity of lung injury [4].

Pulmonary contusions are relatively common in major trauma events (such as motor vehicle accidents, falls from heights, and assaults) [5] but are rarely diagnosed in athletes during competition, with less than 10 case reports available for review [2,6,7]. Additionally, while contrecoup injuries are well-described in traumatic brain injuries, limited reports exist describing coup/contrecoup injury patterns in pulmonary contusions, especially in athletes [8]. Given the overall paucity of literature, there are limited recommendations on return to play. Prior studies have shown that trauma patients with occult pulmonary contusions seen on CT scans but not on radiographs at the time of injury had no statistically significant difference in outcomes compared with control patients without pulmonary contusions and are likely to have an uncomplicated recovery [4,9]. Return to play in two previous cases of football players diagnosed with pulmonary contusions occurred with resolution of symptoms and within one week of injury [6,7]. Overall it appears reasonable to allow a progressive return to play once symptoms have resolved [2,10].

## Conclusions

No previous reports have demonstrated a diagnosis of small bilateral pulmonary contusions during an athletic contest with immediate work-up, rapid symptom resolution, and return to same-game competition, as described in this case. Hypoxemia and large pulmonary contusion volume are independent risk factors for poor prognosis and the development of pulmonary complications. Management of pulmonary contusions is typically supportive, with small contusions often requiring minimal intervention. Return-to-play guidelines are limited by an overall lack of literature describing these injuries in athletes but can generally be based on symptom resolution.
